# Prevalence of hospital-acquired pressure injuries in intensive care units of the Eastern Mediterranean region: a systematic review and meta-analysis

**DOI:** 10.1186/s13037-023-00383-8

**Published:** 2024-01-03

**Authors:** Parvaneh Isfahani, Samira Alirezaei, Somayeh Samani, Fateme Bolagh, Azadeh Heydari, Mohammad Sarani, Mahnaz Afshari

**Affiliations:** 1 Department of Health Services Management, School of Public Health, Zabol University of Medical Sciences, Zabol, Iran; 2 Assistant Professor, Research Center for Social Determinants of Health, Saveh University of Medical Sciences, Saveh, Iran; 3 Department of Occupational Health, Instructor of Occupational Health Engineering, School of Public Health, Zabol University of Medical Sciences, Zabol, Iran; 4 Department of Public Health, School of Public Health, Zabol University of Medical Sciences, Zabol, Iran; 5 Assistant Professor of Health Policy, School of Nursing and Midwifery, Saveh University of Medical Sciences, Saveh, Iran

**Keywords:** Hospitals-acquired pressure sores, Adverse events, Preventable complications, Patient safety, Intensive care unit, Eastern Mediterranean region

## Abstract

**Background:**

Hospital-acquired pressure injuries are a major patient safety concern in intensive care units that are considered largely preventable adverse events by adherence to nursing standards of care. The hypothesis of this research was to investigate the prevalence of hospital-acquired pressure injuries in intensive care units (ICUs) of the Eastern Mediterranean Region.

**Methods:**

This study was designed as a systematic review and meta-analysis. All articles published on Pressure ulcer prevalence in the ICUs of hospitals in Eastern Mediterranean Region countries, identified by searching PubMed through MEDLINE, Web of Science, Scopus, and Google Scholar from January 1, 2011, until September 22, 2023. The reference lists of these articles were checked for additional relevant studies. Data were analyzed using the Comprehensive Meta-Analysis Software (v.2.2.064).

**Results:**

A total of 15 articles met the inclusion criteria. Based on the random-effects model, the overall Pressure ulcer prevalence rate was 16.6% (95% CI (8.6–29.6)). Both the highest and lowest prevalence was observed in Jordan in 2011 at 83.1% (95% CI (71.2- 90.7)) and in 2012 at 0.9% (95% CI (0.5- 1.5)), respectively. The results showed that publication year, average age, and sample size were the main causes of heterogeneity between the reviewed studies (*p* < 0.05).

**Conclusion:**

This systematic review and meta-analysis of the pertinent peer-reviewed literature revealed a high prevalence of hospital-acquired pressure injuries of 16% in intensive care units of Eastern Mediterranean region. Therefore, it is necessary for health policymakers and managers in Eastern Mediterranean Region to take necessary measures to prevent the incidence of Pressure ulcers in hospitals, especially in ICUs.

## Introduction

Healthcare-associated infections are 20 times higher in some developing countries than in developed countries [1–4]. The results of a study on six developing countries, including Egypt, Jordan, Morocco, Sudan, Tunisia and Yemen, showed that about 18% of inpatient admissions were related to adverse events [5].

Pressure ulcers are one of the most common problems faced globally in health care settings [6]. Pressure ulcer or bed sore refers to localized damage to the skin and/or underlying soft tissue, caused by the compression of skin in different parts of the body against a bed, chair or other hard objects. More specifically, the pressure exerted on the tissue reduces blood supply to the skin, leading to the thinning of the epidermis, reduction of subcutaneous fat, and loss of collagen elasticity [7].

Despite technological advances and preventive measures, Pressure ulcers remain a major concern worldwide. The results of a 2020 study showed that in the period 2008–2018, Pressure ulcer prevalence was 12.8% worldwide, 14.5% in Europe, 13.6% in North America, 12.7% in South America, 3% in Asia, 12.6% in the Middle East, and 9% in Australia [8]. Pressure ulcer prevalence in the Eastern Mediterranean Region has varied between 7% and 44.4% [9].

Both intrinsic factors (age, nutritional status, chronic diseases, inactivity, length of stay in the ICU, immune system, radiation therapy, and mental and psychological state) and extrinsic factors (pressure, friction, duration of pressure/friction, skin abrasion, tension, temperature, humidity, trauma, swelling, infection, quality of nursing care, patient repositioning, bed position, and socioeconomic status) play a role in the incidence of Pressure ulcers [10, 11].

Pressure ulcers have many adverse effects on patients, service providers, and the society. Pain caused by Pressure ulcers is one of the most common complaints that causes patient suffering and reduces their quality of life [12, 13]. In addition, they can increase the patient's length of stay and increase the workload of health professionals by causing hospital-acquired infections and disrupting the healing process [14–16]. A 2011 study in Germany showed that patients with Pressure ulcers had a longer stay compared to those without Pressure ulcers (19 days versus 9.9 days). In this study, injuries caused by Pressure ulcers increased unnecessary length of stay by 2.6 days. Longer hospital stays and hospital-acquired infections in turn lead to higher mortality rates, with around 60,000 patients worldwide dying as a result of Pressure ulcers every year [17, 18].

Injuries caused by Pressure ulcers are the third most expensive conditions after cancer and cardiovascular diseases, accounting for approximately 4% of the annual health care budget in Europe [14]. For example, a 2018 study in the US showed that the cost of patients with Pressure ulcers was 22.5% higher than that of other patients [19].

In recent years, several studies have been conducted on Pressure ulcer prevalence in ICUs in various Eastern Mediterranean Region countries, each providing part of the picture of the prevalence of Pressure ulcer across the Eastern Mediterranean Region. For example, a Saudi Arabian study reported an acute care Pressure ulcer prevalence of 44.4% and an incidence of 38.6% [20]. A Jordanian study reported an overall Pressure ulcer prevalence of 12% in the health care setting and 29% in the intensive care setting [21].

However, these studies cannot provide a more complete picture for the entire Eastern Mediterranean Region. Therefore, it is necessary to synthesize the results of the studies conducted to help health managers and officials make evidence-based decisions. Therefore, the purpose of this study was to conduct a systematic review and meta-analysis of studies on Pressure ulcer prevalence in ICUs in hospitals across the Eastern Mediterranean Region.

### Material and methods

The present study is a systematic review and meta-analysis.

### Eligibility criteria

Studies were included in this research if they:

1. Measured prevalence of pressure ulcers in special wards of hospitals.
2. Reported data necessary to calculate it.
3. Were written in English

Studies were excluded if:

- 1. They were thesis, case series, randomized controlled trials, case-control, commentaries, letters to the editor, book chapters, books, editorials, expert opinions, brief reports, and reviews.

### Information sources and search

PubMed through MEDLINE, Web of Science, Scopus, and Google Scholar were searched until 22 September 2023. Search terms included "bed sore", "pressure sore", "pressure ulcer", "decubitus ulcer", hospital, Afghanistan, Bahrain, Djibouti, Egypt, Iran, Iraq, Jordan, Kuwait, Lebanon, Libya, Morocco, Oman, Pakistan, Qatar, Saudi Arabia, Somalia, Sudan, Syrian Arab Republic, Tunisia, United Arab Emirates, Yemen and Palestine by using the AND/OR operators. The electronic search was complemented by hand-searching of the related articles as well as the reference lists of the final studies (Table 1).

**Table 1:** Search stages

Databases	Search strategy	Preliminary searches
PubMed	(("bed sore"[All Fields] OR "pressure sore"[All Fields] OR "pressure ulcer"[All Fields] OR "decubitus ulcer"[All Fields]) AND ("hospital s"[All Fields] OR "hospitalisation"[All Fields] OR "hospitalization"[MeSH Terms] OR "hospitalization"[All Fields] OR "hospitalising"[All Fields] OR "hospitality"[All Fields] OR "hospitalisations"[All Fields] OR "hospitalised"[All Fields] OR "hospitalizations"[All Fields] OR "hospitalized"[All Fields] OR "hospitalize"[All Fields] OR "hospitalizing"[All Fields] OR "hospitals"[MeSH Terms] OR "hospitals"[All Fields] OR "hospital"[All Fields]) AND ("afghanistan"[MeSH Terms] OR "afghanistan"[All Fields] OR "afghanistan s"[All Fields] OR ("bahrain"[MeSH Terms] OR "bahrain"[All Fields]) OR ("djibouti"[MeSH Terms] OR "djibouti"[All Fields]) OR ("egypt"[MeSH Terms] OR "egypt"[All Fields] OR "egypt s"[All Fields]) OR ("iran"[MeSH Terms] OR "iran"[All Fields]) OR ("iraq"[MeSH Terms] OR "iraq"[All Fields]) OR ("jordan"[MeSH Terms] OR "jordan"[All Fields]) OR ("kuwait"[MeSH Terms] OR "kuwait"[All Fields] OR "kuwait s"[All Fields]) OR ("lebanon"[MeSH Terms] OR "lebanon"[All Fields] OR "lebanon s"[All Fields]) OR ("libya"[MeSH Terms] OR "libya"[All Fields]) OR ("morocco"[MeSH Terms] OR "morocco"[All Fields]) OR ("oman"[MeSH Terms] OR "oman"[All Fields]) OR ("pakistan"[MeSH Terms] OR "pakistan"[All Fields] OR "pakistan s"[All Fields]) OR ("qatar"[MeSH Terms] OR "qatar"[All Fields] OR "qatar s"[All Fields]) OR ("saudi arabia"[MeSH Terms] OR ("saudi"[All Fields] AND "arabia"[All Fields]) OR "saudi arabia"[All Fields]) OR ("somalia"[MeSH Terms] OR "somalia"[All Fields]) OR ("sudan"[MeSH Terms] OR "sudan"[All Fields] OR "sudans"[All Fields] OR "sudan s"[All Fields]) OR ("syria"[MeSH Terms] OR "syria"[All Fields] OR ("syrian"[All Fields] AND "arab"[All Fields] AND "republic"[All Fields]) OR "syrian arab republic"[All Fields]) OR ("tunisia"[MeSH Terms] OR "tunisia"[All Fields]) OR ("united arab emirates"[MeSH Terms] OR ("united"[All Fields] AND "arab"[All Fields] AND "emirates"[All Fields]) OR "united arab emirates"[All Fields]) OR ("yemen"[MeSH Terms] OR "yemen"[All Fields]) OR "Palestin"[All Fields])) Filters: Free full text, English, from 1000/1/1—2023/9/22	95
Scopus	ALL ( ″bed AND sore″ OR "pressure sore" OR "pressure ulcer" OR "decubitus ulcer") AND ALL ( hospital) AND ALL ( afghanistan OR bahrain OR djibouti OR egypt OR iran OR iraq OR jordan OR kuwait OR lebanon OR libya OR morocco OR oman OR pakistan OR qatar OR saudi AND arabia OR somalia OR sudan OR syrian AND arab AND republic OR tunisia OR united AND arab AND emirates OR yemen OR palestin) AND ( LIMIT-TO ( DOCTYPE, "ar")) AND ( LIMIT-TO ( LANGUAGE, "English"))	1
Web of Science	(ALL = (″bed sore″ OR "pressure sore" OR "pressure ulcer" OR "decubitus ulcer") AND ALL = (hospital) AND ALL = (Afghanistan OR Bahrain OR Djibouti OR Egypt OR Iran OR Iraq OR Jordan OR Kuwait OR Lebanon OR Libya OR Morocco OR Oman OR Pakistan OR Qatar OR Saudi Arabia OR Somalia OR Sudan OR Syrian Arab Republic OR Tunisia OR United Arab Emirates OR Yemen OR Palestin)) and Open Access and Article (Document Types) and English (Languages)	76
Google Scholar	(″bed sore″ OR "pressure sore" OR "pressure ulcer" OR "decubitus ulcer") AND “special ward” AND (Afghanistan OR Bahrain OR Djibouti OR Egypt OR Iran OR Iraq OR Jordan OR Kuwait OR Lebanon OR Libya OR Morocco OR Oman OR Pakistan OR Qatar OR Saudi Arabia OR Somalia OR Sudan OR Syrian Arab Republic OR Tunisia OR United Arab Emirates OR Yemen OR Palestin)	315

### Study selection process

Search results were imported and managed via EndNote X8 (Thomson Reuters, New York, USA). Duplicates were firstly removed electronically and then manually. Subsequently, the title and abstract of the included studies were independently screened by two reviewers (PI and FB), and disagreements were finally resolved by helping a third reviewer (MH). Full-text of potential studies were retrieved and reviewed by the two reviewers. In order to obtain inaccessible full-texts or English version of the included papers, email or ResearchGate contact was made by the authors.

### Data extraction process

A data extraction sheet was designed and tested by all authors. Two reviewers (PI, MA and FB) extracted data for the country where the study was conducted.

### Data items

Data regarding the following items was collected: the name of the first author, year done, average of age, sample size, special ward, and prevalence of pressure ulcers, and an Excel spreadsheet was used for data entry (Table 2). Primary outcome was the overall prevalence rate of pressure ulcers, secondary outcome come Subgroup analyses (Income level of the country, countries, and type of special ward of the hospital) of the included studies.

**Table 2:** Characteristics of the included studies

**Number**	**Author**	**Year**	**Place**	**Total sample**	**Sample with** Pressure ulcer	**Prevalence (%)**	**Age average**	**Ward**	**Income level**	**Quality article**	**Reference**
1	Al-Ashhab	2011	Jordan	58	28	48.2	_	ICU	lower-middle income	10	[22]
2	Akbari Sari	2014	Iran	90	24	26.7	_	ICU	Lower middle income	13	[23]
3	Azimian	2016	Iran	82	27	32.9	60.9	cardiac intensive care unit	Lower middle income	14	[24]
4	El-Marsi	2014	Lebanon	145	49	33.7	65.0	medical-surgical intensive care unit	lower-middle income	13	[25]
5	Iranmanesh	2011	Iran	82	11	13.4	41.4	trauma intensive care unit	Lower middle income	11	[26]
6	Qaddumi	2017	Palestine	109	36	33	54.7	ICU	Lower middle income	13	[27]
7	Qaddumi	2018	Palestine	140	42	30	_	ICU	Lower middle income	14	[28]
8	Khoshfetrat	2017	Iran	781	71	9.1	_	ICU	Lower middle income	12	[29]
9	Amirah	2015	Saudi Arabia	431	154	35.7	_	ICU	High income	13	[30]
10	Abkenar	2018	Iran	368	126	34.23	Less than a month	NICU	Lower middle income	11	[31]
11	Fallahi	2021	Iran	240	64	26.7	56.26	ICU	Lower middle income	12	[32]
12	Zarei	2019	Iran	643	57	8.9	52.4	ICU	Lower middle income	13	[33]
13	Shokati Ahmadabad	2016	Iran	70	32	45.7	63.4	Open heart cardiac surgery intensive care unit	Lower middle income	13	[34]
14	Al-Wahsh	2012	Jordan	1562	220	14.1	_	ICU	lower-middle income	10	[35]
15	Tayyib	2016	Saudi Arabia	84	33	39.3	52. 20	ICU	High income	13	[9]

### Quality assessment

The methodological quality of the eligible studies was assessed using the 5 questions instrument which introduced and applied by Mitton et al. [36]. Each question was given a score of 0 (not present or reported), 1 (present but low quality), 2 (present and mid-range quality), or 3 (present and high quality). Criteria for assessment of quality included literature review and identifying of research gaps; research questions, hypotheses, and design; population and sampling; data collection process and instruments; and analysis and reporting of results. The assessment was conducted by both MA and SS and discrepancies were then resolved either by discussion or by the third reviewer (MH).

### Summary measures and synthesis of results

Data were analyzed via the Comprehensive Meta-Analysis software (Version 2.2.064). Cochran’s Q-test and I^2^ index were used to test heterogeneity. The I^2^ index was 97.56%, indicating the heterogeneity of the studies. Therefore, a random-effects model was used in this meta-analysis. The effect of variables that could be the potential sources of heterogeneity was examined using the met regression technique. Finally, by using the met regression function, the effect of variables, which potentially accounted for the heterogeneity in the included studies, was examined. The point estimate of the prevalence of pressure ulcers was calculated at the 95% confidence interval (CI) in forest plots, where the size of the box indicates the weight of each study, and the horizontal line indicates the 95% CIs.

## Results

### Study selection

**Fig. 1:**
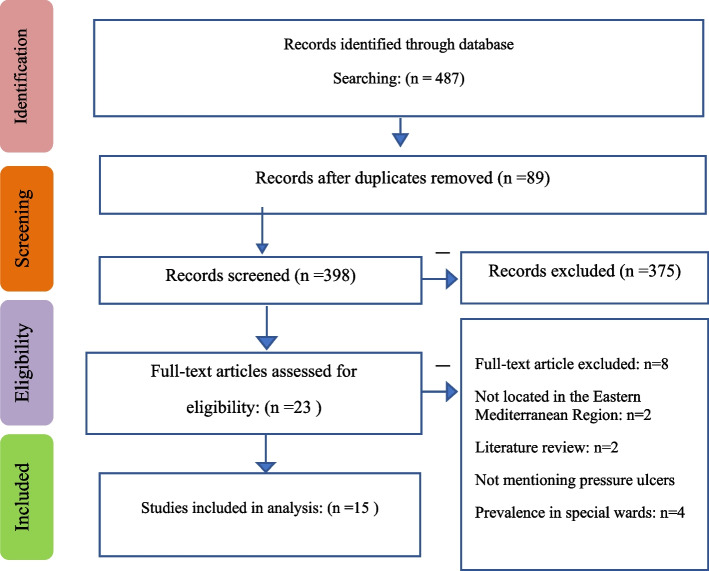
Flowchart of selection and review of articles based on the PRISMA statement

The study selection process and reasons for exclusion are presented in a PRISMA diagram flow  [37]. The initial search resulted in 487 articles. After excluding duplicates and irrelevant articles, 89 studies were selected for abstract examination. After reviewing the abstracts, 375 articles were removed. Also, 8 articles were removed after examining the full texts. Finally, 15 studies were found eligible for inclusion in this systematic review and meta-analysis. Figure 1 demonstrates the search process.

### Study characteristics

**Fig. 2:**
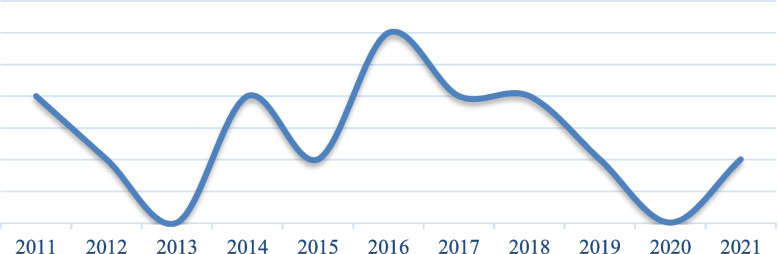
Frequency distribution of reviewed articles by year

A total of 15 articles have determined Pressure ulcer prevalence in ICUs in Eastern Mediterranean Region hospitals from January 1, 2011, until September 22, 2023. Most of the articles have been published in 2016 (Fig. 2). These studies have been conducted in 5 countries, mostly in Iran (8 cases).

The sample size varied from 58 [23] to 1562 [9] hospitals.

**Fig. 3:**
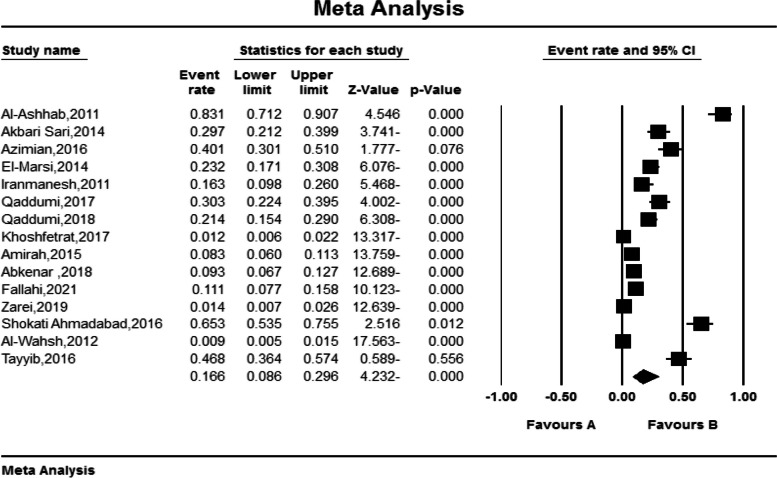
Forest plot of the included studies

Based on the random-effects model, the overall prevalence rate of pressure ulcers was 16.6% (95% CI (8.6–29.6)). The lowest prevalence was observed in Jordan in 2012 at 0.9% (95% CI (0.5- 1.5)), and the highest prevalence was observed in Jordan in 2011 at 83.1% (95% CI (71.2- 90.7)) (Fig. 3).

The results were summarized by Income level of the country, countries, type of special ward of the hospital (Table 3). In this study, pressure ulcers in special wards was more prevalent in Lower-middle income countries than in other countries. Studies in the Open heart cardiac surgery intensive care unit reported higher prevalence rates. Moreover, Pressure ulcers in special wards was more prevalent in Palestine.

**Table 3:** Subgroup analyses of the included studies

variable		Number of studies	Prevalence (%)	95% CI	I^2^	P-value
Countries	High income	2	2.2	0.3–7.2	98.48	≤ 0.0001
	Lower-middle income	13	15.8	7.5–30.5	97.64	≤ 0.0001
Ward	ICU	10	12.6	4.9–28.7	97.97	≤ 0.0001
	Open heart cardiac surgery intensive care unit	1	65.3	53.5–75.5	-	-
	NICU	1	9.3	6.7–12.7	-	-
	Cardiac Intensive Care Unit	1	40.1	30.1–51	-	-
	Medical-Surgical Intensive Unit	1	23.2	17.1–30.8	-	-
	Trauma Intensive Care Unit	1	16.3	9.8–26	-	-
Countries	Iran	8	13	4.9–30	97.42	≤ 0.0001
	Jordan	2	17.4	0–99	99.50	≤ 0.0001
	Lebanon	1	23.2	17.1–30.8	-	-
	Palestine	2	25.6	17.9–35.2	60.28	≤ 0.0001
	Saudi Arabia	2	21.9	2.9–72.3	98.48	≤ 0.0001

The results of the heterogeneity test indicated a high level of heterogeneity between the studies (I^2^ = 97.56%, *P* = 0.0001). Therefore, potential sources of heterogeneity were examined using the meta-regression function. The results are displayed in Table 4, indicating that the year of publication, age average and sample size of articles have caused heterogeneity between the reviewed studies (*p* < 0.05). The results of meta-regression, based on the year of study, demonstrated that an increase of one unit per year of study causes a lower incidence of Pressure ulcers in special wards by 0.07 units. Moreover, the Pressure ulcers in special wards decrease by 0.003 as the sample size of articles increases. On the other hand, an increase of one unit age average causes a higher incidence of Pressure ulcers by 0.017 units.

**Table 4:** The results of the heterogeneity test (meta-regression model)

Suspicious Variables	Coefficient	SE	*P*-Value
**Publication Year**	-0.07	0.023	≤ 0.0001
**Sample Size**	-0.003	0.0001	≤ 0.0001
**Age average**	0.017	0.001	≤ 0.0001

## Discussion

The purpose of this study was to determine Pressure ulcer prevalence in the ICUs of hospitals in the Eastern Mediterranean Region. A total of 15 studies covered this subject from January 1, 2011, until September 22, 2023. It must be noted that the vast majority of these studies were conducted in Iran. This may be partly due to the attempts by the Iranian Ministry of Health at increasing patient safety. In addition, patient safety and strategies for improving it have become a key priority for the Iranian government.

The results of this systematic review and meta-analysis showed that the overall Pressure ulcer prevalence in ICU patients in the Eastern Mediterranean Region is 16.6%. It has been argued that Pressure ulcer prevalence should ideally be less than 2% [38], but their incidence has varied between 2.3% and 23.9% in long-term care facilities, between 0.4% and 38% in acute care facilities, between 0 and 17% in home care, and between 0 and 6% in rehabilitative care [39, 40].

According to a WHO report, up to 18% of hospital admissions in the Eastern Mediterranean Region are associated with adverse events and about 3% are associated with an adverse event that is severe enough to cause death or permanent disability. 83% of recorded adverse events are judged to be preventable [41]. With the help of WHO, Eastern Mediterranean Region countries have adopted a similar approach by way of the Patient Safety Friendly Hospital Initiative (PSFHI). This initiative was launched in 2007 by the Eastern Mediterranean Regional Office of WHO to tackle the enormous problem of unsafe healthcare in the region. The PSFHI follows an earlier effort to document the amount of harm inflicted on patients as a result of the healthcare they receive in hospitals [42].

Pressure ulcer prevalence varied in lower middle-income countries such as Palestine, Jordan, Iran, and Lebanon. For example, among these countries, Palestine and Lebanon had the highest Pressure ulcer prevalence, while Iran had the lowest Pressure ulcer prevalence. Moreover, Pressure ulcer prevalence was studied in one upper-middle income country, i.e., Saudi Arabia, and a prevalence rate of 21.9% was obtained. Therefore, hospital managers and policymakers must focus on improving patient safety and reducing Pressure ulcer prevalence in regions that have higher rates of Pressure ulcer prevalence.

It is necessary to use an evidence-based Pressure ulcer prevention program in hospitals. This program can evaluate the risk of Pressure ulcer outbreak and includes systematic evaluation of the skin, mitigation of risk factors, education to patients and families and personnel, and overall evaluation of the program [43]. Therefore, introducing a formal risk assessment program can significantly reduce the incidence and severity of bedsores in a center [44]. In 2008, the Association for Bedsore Prevention, in collaboration with the New Jersey Hospital Quality Institute (NJHCQI), reported a 30% reduction in bedsores in 150 centers that participated in the Bedsore Prevention Program in the first year of implementation, which reached more than 70% in many centers that participated in the second year of implementation [45].

Pressure ulcer prevalence has been shown to be higher in the open-heart cardiac surgery ICUs. However, this finding should be interpreted with caution as there was only one study in this area. The results of this study showed that for one unit increase in average age, Pressure ulcer prevalence in ICUs increases by 0.017%.

Similarly, Tannen and Dassen reported that age and the length of hospital stay are the most important factors contributing to the incidence of Pressure ulcers [46], which is consistent with our findings.

The presence of comorbidities and reduced physical ability in old age can provide the basis for the incidence of Pressure ulcers. In addition, the economic, social, and psychological problems of the elderly play a significant role in their ability to access quality and specialized care for treating diseases and preventing ulcers. Nonetheless, educating patients, families, and staff about Pressure ulcer prevention can be helpful in mitigating the risk of Pressure ulcers.

The results showed that as publication year increases by one year, Pressure ulcer incidence in ICUs decreases by 0.07. In other words, lower Pressure ulcer prevalence has been reported over time. On the other hand, Pressure ulcer incidence in ICUs decreases by 0.003 as the sample size of articles increases. Therefore, it is necessary to ensure that the sample size is representative of the population and to use a suitable and accurate sampling technique.

Hospital managers and staff should determine the prevalence of Pressure ulcers, analyze the causes of their occurrence, and take necessary measures to prevent their recurrence. Managers should develop policies and guidelines to identify Pressure ulcer outbreaks and communicate them to employees for implementation. Information technology helps hospital managers and staff record Pressure ulcer incidence, identify problem areas faster, and then apply corrective measures. The personnel, especially nurses and ward staff, must receive sufficient training on a regular basis.

Hospital managers should prepare ground for reducing Pressure ulcer prevalence among patients by improving workplace safety, promoting a safety culture, improving work processes, training employees, and increasing their well-being, motivation and satisfaction. Unsuitable workplace conditions such as heat, cold, and lack of resources and equipment increase the likelihood of these events. Therefore, managers should create a safe and suitable working environment for employees and provide them with the necessary equipment and supplies. Hospital managers should promote a safety culture that all employees are committed to uphold [2]. The low well-being and quality of work life of health care providers undermines patient safety [46]. Heavy workload and long shifts lead to fatigue among health care providers. Therefore, improving their well-being and satisfaction should be a top priority for managers.

Some of the main limitations of this study were the unavailability of the full text of some articles, the lack of information in some articles, and the lack of uniform distribution of studies across the different regions under investigation. The lack of uniform reporting in the articles was another limitation of this study; for example, in some studies, Pressure ulcer prevalence was not reported separately by gender, ulcer location, and type of ulcer, which limited the analysis of subgroups.

## Conclusion

This systematic review and meta-analysis of the pertinent peer-reviewed literature revealed a high prevalence of hospital-acquired pressure injuries of 16% in intensive care units of Eastern Mediterranean region. In addition to weakening the immune system, Pressure ulcers have physical, mental, social, and financial consequences for patients, so it is necessary to implement Pressure ulcer prevention measures for susceptible patients. In addition, it is recommended to hold workshops and continuous training programs for hospital staff and, in the same line, proper training for patients and their families in order to reduce Pressure ulcer prevalence in health care facilities.

However, due to the limited number of studies measuring Pressure ulcer prevalence among ICU patients and the small sample size of those studies, the results should be interpreted with caution. Therefore, it is recommended to conduct further research throughout the Eastern Mediterranean Region using a quantitative approach. It is also suggested to conduct qualitative studies to complement quantitative studies and obtain a more comprehensive picture of Pressure ulcer prevalence in ICU patients across this region.

## Data Availability

Not applicable.
